# Corticosteroid-sparing therapy: practice patterns among uveitis specialists

**DOI:** 10.1007/s12348-011-0047-5

**Published:** 2011-11-06

**Authors:** Elizabeth Esterberg, Nisha R. Acharya

**Affiliations:** 1 F.I. Proctor Foundation, University of California San Francisco, Room S309, 513 Parnassus Avenue, San Francisco, CA 94143-0412 USA; 2 Department of Ophthalmology, University of California, San Francisco, San Francisco, CA USA

**Keywords:** Uveitis, Treatment, Immunomodulatory therapy, Corticosteroids, Biologics, Survey

## Abstract

**Purpose:**

This study aims to determine uveitis specialists’ practice patterns, preferences, and perceptions of corticosteroid-sparing therapies for the initial treatment of chronic noninfectious uveitis.

**Methods:**

A survey was distributed to the American Uveitis Society and Proctor email listservs in order to restrict the respondents to specialists who likely have extensive experience in the use of immunomodulatory therapy. Topics included effectiveness, usage, and preferences related to seven immunomodulatory treatments.

**Results:**

Among the 45 responders, the majority (59%) had greater than 10 years of experience treating uveitis. Methotrexate was the most commonly used initial therapy for anterior, intermediate, and posterior/panuveitis (85%, 57%, and 37%), and the most preferred for anterior (55%). Mycophenolate mofetil was the most preferred for intermediate (35%) and posterior/panuveitis (42%). Primary reasons not to prescribe a treatment were effectiveness for azathioprine, safety/tolerability for cyclosporine and cyclophosphamide, and a mixture of cost, safety/tolerability, and difficulty of administration for the biologic drugs.

**Conclusions:**

Within the group of highly experienced uveitis specialists, methotrexate is still the most commonly used initial treatment. Although newer biologic drugs are seen as effective, they are not commonly used, or even preferred, as initial corticosteroid-sparing treatment.

## Introduction

Uveitis is a set of conditions characterized by intraocular inflammation and is a significant cause of vision loss in the USA and the world [1, 2]. Some forms of acute uveitis may be effectively treated with short courses of corticosteroids. Conversely, uveitis that is determined to be chronic and non-infectious in nature often requires the introduction of a corticosteroid-sparing immunomodulatory treatment in order to control inflammation and avoid undesirable complications associated with chronic use of high-dose corticosteroids [3, 4]. Current guidelines recommend starting a corticosteroid-sparing treatment if a dose of greater than 10 mg of oral prednisone is required chronically to control inflammation [3].

A number of immunomodulatory therapy classes are currently used to treat uveitis, including antimetabolites, calcineurin inhibitors, alkylating agents, and biologic drugs. With the exception of the antimetabolites methotrexate and azathioprine, all of these drugs have been introduced in the last 25 years. Due to the low prevalence of uveitis, new treatments have historically been integrated into practice as a result of their success in controlling other autoimmune inflammatory disorders and subsequent anecdotal evidence based on small case series published by uveitis specialists. As collective experience builds, additional evidence has become available in the form of larger retrospective cohort studies and a few small clinical trials.

Guidelines for the use of corticosteroid-sparing immunomodulatory treatments have been established fairly recently to aid clinicians in treating uveitis, but they do not dictate a specific algorithm on how immunomodulatory therapies should be used [3]. There is little information available on what treatments are being used as first-line corticosteroid-sparing agents and the reasons why specific therapies are not preferred. This survey aims to capture the practice patterns and perceptions of uveitis specialists concerning first-line corticosteroid-sparing treatment for chronic uveitis.

## Materials and methods

### Survey population

The survey was distributed by email to 205 members of the American Uveitis Society and Proctor Foundation listservs through the use of the web application surveymonkey.com. The first email, containing a link to the survey, was sent on 10/6/09. All responses were anonymous. The American Uveitis Society is a selective group of uveitis specialists. Admission is voted on by an executive committee and requires applicants to commit at least one third of their time to clinical care and/or research involving immunology/inflammation, at least two first or second author publications on immunology/inflammation in peer-reviewed journals in the last 4 years, and two letters of recommendation, with at least one from a member of the American Uveitis Society. The Proctor Foundation listerv is comprised of specialists in ocular inflammatory disease. There is overlap in these two listservs, but each respondent was only allowed to submit one survey response. This population was chosen despite its small size in order to elicit opinions only from uveitis experts who are likely to have extensive experience with the use of immunomodulatory therapies as steroid-sparing treatment.

### Survey

The survey consisted of five sections and included seven immunomodulatory therapies: methotrexate, mycophenolate mofetil, azathioprine, cyclosporine, cyclophosphamide, infliximab, and adalimumab. The first section contained Likert scales for rating the effectiveness of seven therapies for controlling inflammation and allowing a successful corticosteroid taper in three anatomical locations of uveitis (anterior, intermediate, and posterior/panuveitis). Effectiveness ratings were reported on a four-point Likert scale, with 1 and 2 representing unfavorable responses of “Not effective” and “Somewhat effective”, and 3 and 4 representing favorable responses of “Mostly effective” and “Very effective”. The second and third sections captured which treatments are most commonly used as first-line corticosteroid-sparing therapy and which would be preferred in an ideal world where cost and availability were not an issue. In these sections, respondents were asked to rank-order the seven treatments within each anatomic location according to actual use and preference. The fourth section had a variety of questions to determine perceived disadvantages of each treatment, as well as more detailed questions on the administration of methotrexate and what dose levels of systemic and topical corticosteroids are considered acceptable maintenance doses. Finally, demographic information on the respondents was collected. Institutional review board exemption was obtained.

### Statistical methods

Descriptive analyses were conducted with binomial 95% confidence intervals where appropriate. Additional analyses of Likert-style questions were conducted using Kruskal–Wallis analysis of variance to compare effectiveness of treatments [5]. *P* values shown are nominal (16 hypothesis tests were performed) and only referred to as statistically significant when the reported value was less than 0.003 according to the Bonferroni correction for multiple comparisons. Worth estimates were calculated based on ranking data using a Bradley–Terry model to represent what fraction of overall worth could be assigned to each treatment and to estimate an overall ranking [6]. All analyses were conducted using the statistical software R (www.R-project.org).

## Results

### Physician demographics and characteristics

Of the 205 clinicians contacted through the listserv, 45 completed the survey. All respondents characterized themselves as practicing uveitis specialists, of which the majority (59%) had more than 10 years of experience, and an additional 14% had 6–10 years of experience. Sixty-eight percent of respondents practice in a university or academic setting, 27% in a private solo/group practice, and 5% in a health maintenance organization. Ninety-seven percent prescribe and manage immunomodulatory therapy themselves at least some of the time, with 41% reporting that they always manage such treatment themselves. Eighty-three percent of the respondents practice in the USA, 6% in Mexico, 6% in Europe, 3% in Australia, and 3% in Canada.

### Treatment effectiveness

Treatment effectiveness was defined as the ability to control ocular inflammation and successfully taper corticosteroids to an acceptable maintenance dose. According to our respondents, the median acceptable maintenance dose of oral prednisone (i.e., corticosteroid-sparing) was 7.5 mg/day (range 0 to 10 mg/day), and the median acceptable dose of topical prednisolone acetate 1% was 2 drops/day (range 1 to 6 drops/day).

The median efficacy ratings for each corticosteroid-sparing treatment and anatomic location are shown in Table 1. Within each anatomic location, respondents thought there were significant differences between the effectiveness levels of the seven drugs (*P* ≤ 0*.*001). As for perceived differences in the effectiveness of each drug within a specific anatomic location, only methotrexate had a statistically significant difference. Respondents considered methotrexate to be only somewhat effective for treating patients with intermediate, posterior, and panuveitis, but mostly effective for those with anterior uveitis (*P* ≤ 0*.*001). This perceived difference in effectiveness was reflected in the favorability ratings for methotrexate, which were 62% for anterior, 44% for intermediate, and 22% for posterior and panuveitis (Table 2). Adalimumab was considered to be mostly effective for patients with intermediate, posterior, and panuveitis, and very effective for anterior uveitis (*P* = 0.04). Similarly, reported effectiveness ratings for cyclosporine were higher in intermediate and posterior/panuveitis compared to anterior uveitis (*P* = 0.08). Only 20% responded favorably concerning the use of cyclosporine in patients with anterior uveitis, while 38% and 44% assigned a favorable rating for its use in intermediate and posterior/panuveitis, respectively. Infliximab had the highest overall favorability ratings for effectiveness (82%, 69%, 71% for anterior, intermediate, and posterior/panuveitis, respectively) while azathioprine had the lowest (29%, 31%, 33%).

**Table 1:** Median effectiveness ratings^a^ of immunomodulatory treatments

	MTX	MMF	AZA	CSA	CTX	INF	ADA	
Anterior	3	3	2	2	4	4	4	<0.001
Intermediate	2	3	2	2.5	4	3	3	<0.001
Posterior/panuveitis	2	3	2	3	4	3.5	3	<0.001
	<0.001	0.44	0.79	0.08	0.87	0.47	0.04	*P* value

*MTX* methotrexate, *MMF* mycophenolate mofetil, *AZA* azathioprine, *CSA* cyclosporine, *CTX* cyclophosphamide, *INF* infliximab, *ADA* adalimumab

^a^Respondents were asked to rate the effectiveness of each immunomodulatory therapy by anatomic subtype on a four-point scale (1 = not effective, 2 = somewhat effective, 3 = mostly effective, 4 = very effective)

**Table 2:** Favorability ratings of immunomodulatory therapies

	MTX	MMF	AZA	CSA	CTX	INF	ADA
Favorable^a^							
Anterior	28 (62%)	24 (53%)	13 (29%)	9 (20%)	21 (47%)	37 (82%)	33 (73%)
Intermediate	20 (44%)	27 (60%)	14 (31%)	17 (38%)	24 (53%)	31 (69%)	26 (58%)
Posterior/panuveitis	10 (22%)	24 (53%)	15 (33%)	20 (44%)	32 (71%)	32 (71%)	25 (56%)
No opinion							
Anterior	0 (0%)	7 (16%)	19 (42%)	16 (36%)	20 (44%)	5 (11%)	10 (22%)
Intermediate	1 (2%)	4 (9%)	13 (29%)	11 (24%)	17 (38%)	7 (16%)	12 (27%)
Posterior/panuveitis	1 (2%)	2 (4%)	11 (24%)	7 (16%)	9 (20%)	7 (16%)	16 (36%)

*MTX* methotrexate, *MMF* mycophenolate mofetil, *AZA* azathioprine, *CSA* cyclosporine, *CTX* cyclophosphamide, *INF* infliximab, *ADA* adalimumab

^a^Rating question responses of “mostly effective” or “very effective” were considered favorable. Respondents could also indicate if they had no opinion for a particular combination

Approximately 60% of respondents believe that methotrexate is more effective when administered subcutaneously compared to by mouth. However, of patients prescribed methotrexate doses of 20 mg/week and 25 mg/week, only an average of 19% and 25%, respectively, were placed on subcutaneous treatment. The mean maintenance dose of methotrexate used was 18.5 mg weekly (range 7.0 to 25.0 mg weekly).

### Used vs. preferred immunomodulatory therapy

**Fig. 1:**
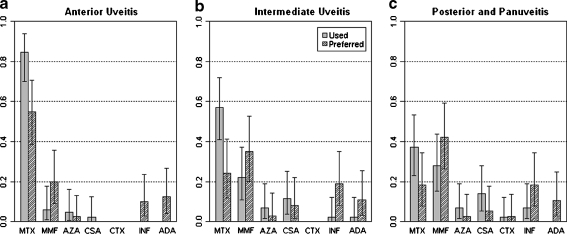
Histograms showing proportions of respondents, with 95% onfidence intervals, ranking each treatment as most commonly used or most preferred if cost and availability were not an issue. *MTX* methotrexate, *MMF* mycophenolate mofetil, *AZA* azathioprine, *CSA* cyclosporine, *CTX* cyclophosphamide, *INF* infliximab, *ADA* adalimumab

The majority of respondents reported that methotrexate was their *most commonly used* initial corticosteroid-sparing treatment for noninfectious uveitis in all three anatomic subgroups, followed by mycophenolate mofetil (85% vs. 6% for anterior, *P* < 0.001; 57% vs. 22% for intermediate, *P* = 0.002; 37% vs. 27% for posterior/panuveitis, *P* = 0.49) (see Fig. 1). Azathioprine, cyclophosphamide, and infliximab were rarely or never used as initial corticosteroid-sparing treatment, and none of the respondents listed adalimumab as being used for initial treatment for any anatomic subgroup.

When asked about a scenario where cost and availability of therapies were not an issue, 55% still reported that methotrexate was their *most preferred* initial corticosteroid-sparing treatment for anterior uveitis, followed by 20% for mycophenolate mofetil (*P* = 0.003). For intermediate and posterior/panuveitis, however, mycophenolate mofetil was most frequently preferred, followed by methotrexate (35% vs. 24% for intermediate, *P* = 0.45; 42% vs. 18% for posterior/panuveitis, *P* = 0.04). Some respondents reported that they would prefer to use infliximab (10% anterior, 19% intermediate, 18% posterior/panuveitis) and adalimumab (13% anterior, 11% intermediate, 11% posterior/panuveitis) as initial treatment in each subgroup. Worth estimates based on ranking data indicate that methotrexate and mycophenolate mofetil are consistently ranked first or second overall in all scenarios, while cyclosporine, azathioprine, adalimumab, and infliximab are clustered in the middle and cyclophosphamide is consistently ranked last. The exact order of treatment use and preference fluctuated depending on anatomic location (Table 3).

**Table 3:** Overall rankings by anatomic location of uveitis and corresponding worth estimates^a^

Rank	Anterior		Intermediate		Posterior/pan	
	Used	Preferred	Used	Preferred	Used	Preferred
1	MTX (0.60)	MTX (0.30)	MTX (0.24)	MMF (0.23)	MMF (0.21)	MMF (0.22)
2	MMF (0.13)	MMF (0.22)	MMF (0.21)	MTX (0.20)	MTX (0.19)	MTX (0.18)
3	INF (0.07)	INF (0.14)	AZA (0.13)	INF (0.16)	AZA (0.14)	INF (0.15)
4	AZA (0.07)	ADA (0.13)	CSA (0.13)	ADA (0.13)	CSA (0.14)	ADA (0.15)
5	ADA (0.06)	AZA (0.10)	INF (0.13)	AZA (0.12)	INF (0.13)	CSA (0.12)
6	CSA (0.05)	CSA (0.07)	ADA (0.11)	CSA (0.11)	ADA (0.11)	AZA (0.11)
7	CTX (0.02)	CTX (0.04)	CTX (0.05)	CTX (0.05)	CTX (0.08)	CTX (0.07)

*MTX* methotrexate, *MMF* mycophenolate mofetil, *AZA* azathioprine, *CSA* cyclosporine, *CTX* cyclophosphamide, *INF* infliximab, *ADA* adalimumab

^a^Calculated using a Bradley–Terry model: represent what fraction of overall worth could be assigned to each treatment and are used to estimate an overall ranking (e.g., based on the collective rankings of the respondents, methotrexate could be assigned 60% of the collective preference as the most used initial treatment for anterior uveitis)

### Reasons immunomodulatory therapies are not prescribed

**Fig. 2:**
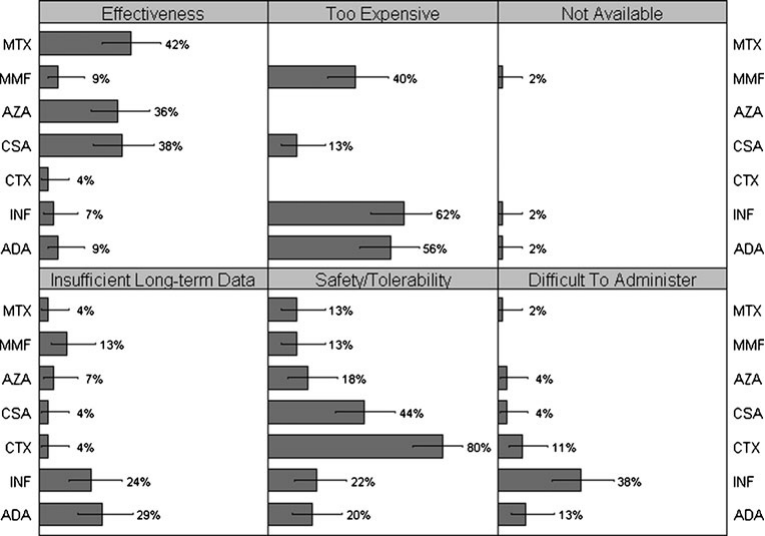
Histogram annotated with percentage of respondents, and 95% confidence intervals, citing each reason for not prescribing specific immunomodulatory therapies. *MTX* methotrexate, *MMF* mycophenolate mofetil, *AZA* azathioprine, *CSA* cyclosporine, *CTX* cyclophosphamide, *INF* infliximab, *ADA* adalimumab

Respondents indicated that they might choose not to prescribe each of the drugs for different reasons (Fig. 2). The most common reason not to prescribe methotrexate or azathioprine was concern about effectiveness (42% and 36%, respectively), although there was also some concern about safety/tolerability (13% and 18%). Common reasons not to prescribe mycophenolate mofetil included cost (40%), lack of long-term data on its use (13%), and safety/tolerability (13%). The primary concern with using cyclosporine or cyclophosphamide was safety/tolerability (44% and 80%), but other reasons not to prescribe cyclosporine included effectiveness (38%) and cost (13%). The most common reason not to prescribe infliximab and adalimumab was cost (62% and 56%, respectively). Other prominent concerns for these biologic drugs included a lack of long-term data (24% and 29%), safety/tolerability (22% and 20%), and difficulty of administration (38% and 13%).

## Discussion

Our results indicate that within each anatomic location, there are significant differences in the perceived effectiveness of corticosteroid-sparing treatments for controlling ocular inflammation and allowing a successful corticosteroid taper.

Though they do not receive the highest favorability ratings for effectiveness, the most commonly used and most preferred drugs are from the antimetabolite class. Respondents indicate that the long-used methotrexate is their most common first choice corticosteroid-sparing therapy for all anatomic locations of uveitis, with only mild concerns for safety and tolerability. Even given the choice of any other drug disregarding cost and availability, most would still prefer to prescribe methotrexate for anterior uveitis. Methotrexate, however, was the only drug in which there was a significant difference in effectiveness ratings by anatomic location. For intermediate and posterior/panuveitis, respondents would prefer to use mycophenolate mofetil; the main reason for not prescribing mycophenolate initially seems to be cost. These trends were seen when looking at first choices for most commonly used and most preferred treatments and when evaluating the full ranking data using a Bradley–Terry model. This is a well-developed method of rank-ordering items based on how each respondent ranks each item in relation to the others, and may provide a more relevant estimate of the relative usage and preference within our survey population by incorporating more in-depth information [6].

Even with the recent availability of generic mycophenolate mofetil, the cost per month is still more than double that of methotrexate. According to Medicare reimbursement rates, a 1-month supply of maintenance dose methotrexate (25 mg per week) costs $50.55, and an equivalent supply of generic mycophenolate mofetil (1 g twice a day) costs $118.50 [7]. Retrospective studies by the Systemic Immunosuppressive Therapy for Eye Diseases Cohort Study Research Group, the largest such studies to date, have reported corticosteroid-sparing success rates at 6 months with methotrexate and mycophenolate for posterior/panuveitis at 21% and 41%, respectively [8, 9]. Another study, comprised mostly of patients with posterior/panuveitis, reported success rates of 42% with methotrexate compared to 79% with mycophenolate mofetil [10]. There have been no controlled trials comparing methotrexate and mycophenolate mofetil for any anatomic location to confirm the differences found in retrospective studies. Azathioprine, the third antimetabolite option, was the least popular of the three among our respondents. Published retrospective studies report similar effectiveness rates to those of methotrexate for uveitis, but discontinuations due to safety and tolerability may be more frequent [9–12]. Azathioprine is also widely used in rheumatologic diseases and organ transplantation. Randomized trials comparing methotrexate to azathioprine have shown similar effectiveness for treating ANCA-associated vasculitis [13] and myasthenia gravis [14], and mixed results in rheumatoid arthritis with one small trial showing similar effectiveness [15] and another showing greater effectiveness with methotrexate [16]. Mycophenolate mofetil was shown to be more effective than azathioprine in a randomized trial of patients with Crohn’s disease [17] and for cardiac transplantation [18], but trials in lupus nephritis [19] and renal transplantation [20] found no significant differences.

Despite being the least used and least preferred as first-line corticosteroid-sparing treatment, the biologic drugs adalimumab and infliximab and the alkylating agent cyclophosphamide received the overall highest effectiveness ratings based on both medians and percent of respondents giving a favorable response. Actual use and preference, however, do not match these reported beliefs. In fact, adalimumab, infliximab, and cyclophosphamide are infrequently used and preferred as initial corticosteroid-sparing treatment, even if cost and availability are not an issue. In the case of adalimumab and infliximab, respondents cited a number of reasons not to prescribe these treatments, including cost, insufficient long-term data, concerns with safety and tolerability, and difficulty of administration. Adalimumab is given by subcutaneous injections and infliximab by intravenous infusions. Despite the overwhelming opinion that cyclophosphamide is effective, it was the least preferred drug, primarily due to concern for the safety of the patient. Cyclophosphamide has been associated with an increased risk of malignancy, infertility, and other undesirable side effects, so this finding was not surprising [21]. Cyclosporine, a calcineurin inhibitor, was not commonly used and rarely preferred as first-line corticosteroid-sparing therapy because of safety and tolerability concerns and some doubt as to its effectiveness. Previous publications report widely varying rates of success with cyclosporine for uveitis, and there is evidence of high rates of side effects including nephrotoxicity [22].

This study does have limitations. It is possible that those who chose to participate in the survey are somehow different from those who did not, which would affect the generalizability of our results to all uveitis specialists or even to the members of the listservs as a whole. The sample size raises the question of whether the responses collected in this study reflect actual practice patterns of uveitis specialists, though studies have shown surveys with similar response rates (about 25%) to have comparable results to those with higher response rates [23–25]. Increased sample size could have been achieved by including a broader sample of ophthalmologists in the study, but this would have potentially compromised our goal of eliciting opinions from uveitis specialists with extensive experience with immunomodulatory therapy. The respondents were all uveitis specialists, and the majority had greater than 10 years of practice experience, likely indicating the respondent group had a high level of expertise in this subject area. This is also highlighted by the fact that 100% of respondents reported maximum oral corticosteroid doses of 10 mg/day or less as acceptable maintenance levels, demonstrating familiarity with the SUN guidelines on the use of immunosuppressives [3]. This is in contrast with the population of a recent survey focusing on corticosteroid use in which steroid-sparing immunosuppressives were rarely used and an average prednisone maintenance dose of 34 mg/day was reported [26]. In addition, as the vast majority of the respondents in our survey practice in the USA, reported practice patterns and preferences may be specific to the USA and could be different in other countries.

Perceptions of effectiveness and negative aspects of each treatment could be affected in a number of ways. Although general guidelines for the use of immunomodulatory treatment of noninfectious uveitis have been established, uveitis specialists may differ in the way they use each treatment (varying maintenance doses, etc). They may also treat patients with varying disease severities and etiologies of uveitis, which could affect perceptions about each treatment. It is also important to note that this survey reported usage and preferences based on anatomical location of inflammation rather than any associated disease entity. For patients with severe uveitis-related complications or a known associated inflammatory disease such as juvenile idiopathic arthritis or Behcet’s, the nature of the results may have been different. Other factors such as co-management with a rheumatologist and age of the patient population may also affect practice patterns.

Additionally, not all immunomodulatory drugs currently used to treat uveitis were included in the survey; omitted treatments include the calcineurin inhibitors tacrolimus and sirolimus, the alkylating agent chlorambucil, and newer biologic agents such as golimumab or certolizumab. Etanercept was also not included; though etanercept has been commercially available since 1999 and is used to treat various systemic inflammatory conditions, a number of studies have indicated that this particular TNF-alpha inhibitor is likely not effective for controlling ocular inflammation [27–29]. There was initially some concern in the rheumatology literature that etanercept may even induce ocular inflammation, but more recent findings support the continued use of etanercept therapy for inflammatory diseases with the caveat that patients developing uveitis may require a change in treatment regimen [30]. We chose to include treatments which have been most commonly reported in the literature to ensure that most respondents would have some experience with each, making comparisons between them possible.

Despite potentially mitigating factors, the results of this survey were striking. They raise questions that warrant further study, including the possibility of variable effectiveness by anatomic location for some treatments, and also highlight factors that uveitis specialists feel limit the practical use of each immunomodulatory treatment. These results may help guide future research comparing treatment effectiveness for initial corticosteroid-sparing therapy in noninfectious uveitis.
